# Diagnostic performance of standardized prenatal ultrasound signs for predicting Hecht histopathological grade in placenta accreta spectrum: a retrospective study

**DOI:** 10.3389/fmed.2026.1933234

**Published:** 2026-09-18

**Authors:** Matus Halaj, Petra Hanulikova, Sona Stankova-Lisova, Lubomir Haslik, Hynek Herman, Blanka Prosova, Ladislav Krofta

**Affiliations:** 1 Department of Gynecology and Obstetrics, Institute for the Care of Mother and Child, Prague, Czechia; 2 Third Faculty of Medicine, Charles University, Prague, Czechia; 3 Department of Radiology, Motol and Homolka University Hospital, Prague, Czechia; 4 Second Faculty of Medicine, Charles University, Prague, Czechia

**Keywords:** cesarean delivery, Hecht classification, maternal morbidity, peripartum hemorrhage, placenta accreta spectrum, prenatal ultrasound

## Abstract

**Objective:**

To evaluate the diagnostic performance of prenatal ultrasound in predicting histopathological grade of placenta accreta spectrum (PAS) and to assess associated maternal and neonatal outcomes.

**Methods:**

We performed a retrospective cohort study based on prospectively collected standardized prenatal ultrasound data, including patients with suspected PAS referred to a tertiary center between 2018 and 2025. Eligible patients underwent standardized prenatal ultrasound assessment and delivery at our institution with histopathological evaluation. Ultrasound signs included myometrial thinning, loss of the clear zone, placental lacunae, utero-vesical hypervascularity (bridging vessels), abnormal placental–uterine interface, and placental bulging. Patients were categorized into low-grade (Hecht grade ≤2 or BPMF) and high-grade (Hecht grade ≥3A) groups. Diagnostic accuracy measures were calculated for each sign.

**Results:**

Forty patients met inclusion criteria (15 low-grade, 25 high-grade). Diagnostic performance was assessed in the 35 women with an assignable Hecht grade. Myometrial thinning and placental lacunae demonstrated the highest sensitivity for predicting high-grade PAS (92.0% each), while bridging vessels and placental bulging showed the highest specificity among the signs (70.0% each). High-grade cases were associated with greater estimated blood loss, lower gestational age at delivery, and lower birth weight. Hysterectomy and internal iliac artery ligation were more frequent in the high-grade group.

**Conclusion:**

In this cohort, prenatal ultrasound assessment showed differential ability to discriminate high-grade PAS confirmed by histopathology. Higher histological grade was associated with increased maternal morbidity.

## 1 Introduction

Placenta accreta spectrum (PAS) represents a severe obstetric condition characterized by abnormal adherence of placental tissue to the myometrium and is associated with substantial maternal morbidity and mortality (1). Historically, the pathophysiology of PAS was attributed to excessive trophoblastic invasion beyond its physiological boundary, suggesting aberrant biological behavior of the trophoblast as the primary mechanism of pathological placental adherence (2).

Current evidence supports a different mechanism centered on defective uterine tissue remodeling, particularly at the site of a prior cesarean section scar. Inadequate decidualization and focal myometrial loss bring anchoring villi into close proximity with the uterine surface, facilitating direct interaction with large maternal arteries. This disruption of normal extravillous trophoblast regulation alters uteroplacental circulation and is often associated with abnormal fibrinoid deposition and impaired spontaneous placental separation at delivery (3, 4).

The underlying pathophysiology of PAS is reflected in its imaging characteristics. Loss of normal decidua and thinning of the myometrium correlate with ultrasound findings such as disappearance of the clear zone, myometrial thinning, and placental bulging. Altered uteroplacental circulation manifests as utero-vesical hypervascularity, bridging vessels, bladder wall interruption, and prominent placental lacunae with feeder vessels (1, 3).

Prenatal diagnosis relies primarily on high-resolution ultrasound, with magnetic resonance imaging (MRI) serving as a complementary modality in selected cases. When performed by experienced operators, imaging findings correlate closely with pathological changes and form the basis of current diagnostic criteria, risk stratification, and surgical planning (5).

The clinical consequences of PAS are significant. Affected women face an increased risk of severe postpartum hemorrhage, hysterectomy, blood transfusion, and maternal death (6, 7).

Although the diagnostic accuracy of ultrasound for antenatal identification of PAS has been widely reported, fewer studies have examined how individual ultrasound signs correlate with histopathological grade, which is directly relevant to surgical complexity and maternal morbidity. Therefore, we aimed (I) to assess the diagnostic performance of individual prenatal ultrasound signs for distinguishing low- versus high-grade PAS using Hecht histopathological grading as the reference standard, and (II) to compare maternal and neonatal outcomes between grade groups. We hypothesized that individual prenatal ultrasound signs would predict histopathological PAS grade according to the Hecht classification, and that high-grade PAS would be associated with increased maternal morbidity and less favorable neonatal outcomes.

## 2 Materials and methods

This was a retrospective cohort study based on prospectively collected standardized prenatal ultrasound data. We included patients with prenatal ultrasound suspicion of placenta accreta spectrum (PAS) referred to a tertiary referral center, who delivered at our institution and underwent histopathological evaluation of the placental or uterine specimen between January 2018 and November 2025.

Since January 2018, patients with suspected PAS at our center have been evaluated using a standardized prenatal ultrasound protocol performed by two fetal-maternal specialists with more than 10 years of expertise in prenatal ultrasound and placental imaging. The protocol was applied uniformly throughout the study period.

The six ultrasound signs analyzed in this study were prospectively assessed and documented in the imaging reports performed between 24 and 32 weeks of gestation (median 29 + 5 weeks), before delivery, surgery, and histopathological evaluation. No retrospective reinterpretation of stored ultrasound images was performed. Examinations were performed transabdominally and transvaginally using Voluson E10 and Voluson Expert 22 (GE HealthCare).

Patients were excluded if delivery occurred outside our institution, if no histopathological specimen was available or was insufficient for grading, or if imaging or clinical documentation was incomplete.

### 2.1 Prenatal ultrasound assessment

The following ultrasound signs were systematically assessed and recorded, based on the FIGO consensus criteria (5):

- **Myometrial thinning**, defined as thinning of the myometrium overlying the placenta to < 1 mm;
- **Loss of the clear zone**, defined as absence or irregularity of the hypoechoic plane between the placenta and the myometrium;
- **Placental bulging**, defined as protrusion of placental tissue beyond the expected uterine contour toward an adjacent organ, most commonly the bladder;
- **Placental lacunae**, defined as multiple irregular intraplacental spaces with turbulent flow on color Doppler imaging;
- **Utero-vesical hypervascularity (bridging vessels)**, defined as vessels extending from the placenta across the uterine serosa toward the bladder;
- **Abnormal placental–uterine interface**, defined as disruption or irregularity of the normal placental–myometrial boundary.

### 2.2 Histopathological evaluation

All placental and uterine specimens were examined by board-certified pathologists with expertise in gynecological pathology. Because the Hecht classification was published in 2020 — during the study period — retrospective grading of the pre-2020 cases (*n* = 14) was performed by board certified pathologists with expertise in placental pathology against these criteria on the basis of the original pathology reports and the archived hematoxylin and eosin–stained slides. Pathologists were blinded to the prenatal ultrasound findings and maternal and neonatal outcomes. Cases delivered from 2020 onward were graded against the Hecht criteria at routine histopathological sign-out. For each case, the area of deepest placental invasion was specifically sampled. In all cases, pathologists were blinded to prenatal ultrasound findings and to intraoperative surgical judgment. The material available for grading differed according to management. In the 29 patients who underwent hysterectomy, the full thickness uterine wall with the placenta *in situ* was available. In the 11 patients managed without hysterectomy, all of whom were classified as low grade, the placenta with underlying myometrium from resected placental bed were available. Because eligibility was based on prenatal suspicion of PAS with subsequent histopathological evaluation, the cohort includes cases in which the findings met criteria for basal plate myometrial fibers (BPMF) rather than an assignable PAS grade; this applied to five women. These cases were analyzed within the low-grade group, since BPMF and grade 1 both denote superficial adherence without deep myometrial invasion. Six cases were excluded because the available material was insufficient for grading. Grading was performed by consensus of the reporting pathologists; formal inter-observer agreement was not quantified. Histopathological grade was classified according to the Hecht classification system (8). For analytical purposes, patients were categorized into two groups:

- Low-grade PAS, defined as Hecht grade ≤2 or BPMF;
- High-grade PAS, defined as Hecht grade ≥3A.

The threshold between grade 2 and grade 3A was chosen *a priori* on clinical grounds. Grade 2 denotes myometrial invasion with substantial residual uterine wall, whereas grade 3A denotes deep invasion leaving less than 25% of the wall thickness intact. The sample size did not allow finer stratification, and no data-driven cut-point was sought.

### 2.3 Outcomes

The **primary outcome** was the diagnostic performance of individual prenatal ultrasound signs in distinguishing low- versus high-grade PAS based on histopathological grading.

**Secondary outcomes** included maternal morbidity, defined as estimated intraoperative blood loss, hysterectomy, internal iliac artery ligation, and gestational age at delivery. Neonatal outcomes included birth weight and Apgar score.

The decision between uterine preservation and hysterectomy was made intraoperatively, after delivery of the fetus and assessment of the extent of invasion and the area involved. In cases confirmed intraoperatively as low grade, conservative management consisted of focal resection of the PAS area or manual placental separation.

Exploratory analyses assessed associations between histopathological grade and known risk factors, including the number of previous cesarean deliveries.

### 2.4 Statistical analysis

Categorical variables were summarized as frequencies and percentages and compared using the chi-square test or Fisher's exact test, as appropriate. Continuous variables were assessed for normality using visual inspection and the Shapiro–Wilk test. As continuous variables were not normally distributed, they are presented as median with interquartile range (IQR) and were compared between low- and high-grade groups using the Mann–Whitney U test. Diagnostic performance of individual ultrasound signs was evaluated by calculating sensitivity, specificity, positive predictive value (PPV), and negative predictive value (NPV) using final histopathological grade as the reference standard. Corresponding 95% confidence intervals were calculated for all measures using the Wilson score method. Ordered categorical associations (number of previous cesarean deliveries vs. grade) were tested using the chi-square test for trend. All tests were two-sided, and a *p* value < 0.05 was considered statistically significant. Because basal plate myometrial fibers (BPMF) are not an assignable Hecht grade, diagnostic performance was calculated in the 35 women with an assignable grade; a sensitivity analysis including the five BPMF cases within the low-grade group (*n* = 40) is reported in Supplementary Table S1. Reporting follows the STARD 2015 checklist for diagnostic accuracy studies; the flow of participants through screening, eligibility, and grading is shown in Figure 1.

**Figure 1:**
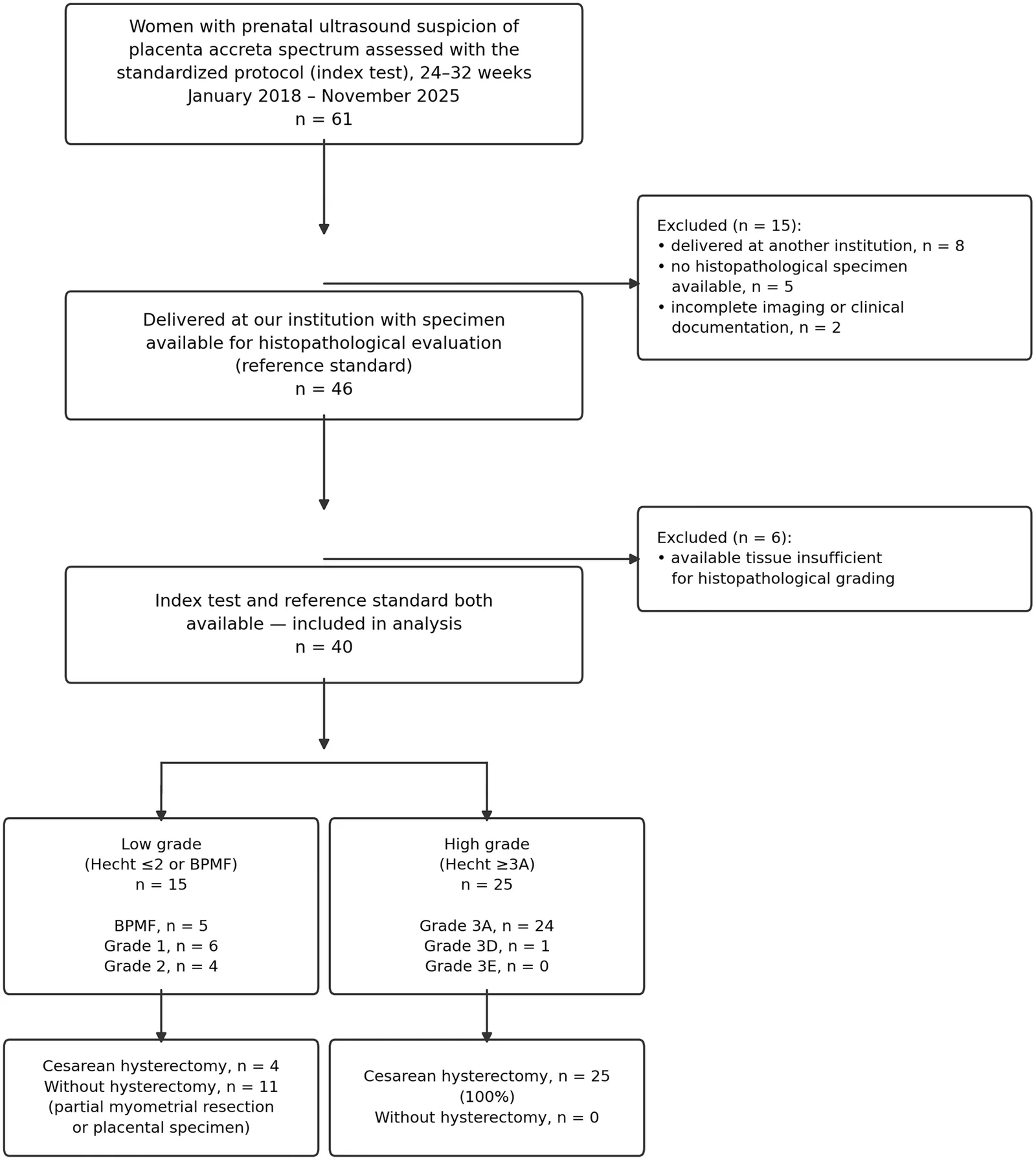
Flow of participants through the study, reported according to STARD 2015. BPMF, basal plate myometrial fibers.

## 3 Results

Of 61 women assessed with the standardized protocol, 40 met inclusion criteria and were analyzed (Figure 1).

The distribution of histopathological findings was: BPMF, *n* = 5; grade 1, *n* = 6; grade 2, *n* = 4; grade 3A, *n* = 24; grade 3D, *n* = 1; grade 3E, *n* = 0. The proportion of high-grade cases in this cohort is higher than would be expected in an unselected obstetric population, reflecting the referral pattern of a tertiary center to which women with a strong prenatal suspicion of PAS are referred for delivery.

The diagnostic performance of individual prenatal ultrasound signs relative to final histopathological grade is summarized in Table 1. Myometrial thinning and placental lacunae showed high sensitivity, whereas placental bulging and bridging vessels demonstrated higher specificity. In a sensitivity analysis including the five BPMF cases (*n* = 40), sensitivity was identical, as the high-grade group was unaffected; specificity was 46.7% for myometrial thinning, 60.0% for loss of the clear zone, 13.3% for placental lacunae, and 73.3% for bridging vessels and placental bulging. The relative ranking of the signs was unchanged (Supplementary Table S1). All five BPMF cases showed placental lacunae.

**Table 1:** Diagnostic performance of ultrasound signs in the 35 women with an assignable hecht grade for identifying high-grade PAS.

Ultrasound sign	Sensitivity, % (95% CI)	Specificity, % (95% CI)	PPV, % (95% CI)	NPV, % (95% CI)
Myometrial thinning (<1 mm)	92.0 (75.0–97.8)	30.0 (10.8–60.3)	76.7 (59.1–88.2)	60.0 (23.1–88.2)
Loss of the clear zone	84.0 (65.3–93.6)	50.0 (23.7–76.3)	80.8 (62.1–91.5)	55.6 (26.7–81.1)
Placental lacunae	92.0 (75.0–97.8)	20.0 (5.7–51.0)	74.2 (56.8–86.3)	50.0 (15.0–85.0)
Bridging vessels	80.0 (60.9–91.1)	70.0 (39.7–89.2)	87.0 (67.9–95.5)	58.3 (32.0–80.7)
Abnormal placental–uterine interface	80.0 (60.9–91.1)	50.0 (23.7–76.3)	80.0 (60.9–91.1)	50.0 (23.7–76.3)
Placental bulging	80.0 (60.9–91.1)	70.0 (39.7–89.2)	87.0 (67.9–95.5)	58.3 (32.0–80.7)

PAS, Placenta Accreta Spectrum; PPV, Positive Predictive Value; NPV, Negative Predictive Value.

Sensitivity, Specificity, PPV, and NPV were calculated against the final histological grade (*N* = 10 low grade, *N* = 25 high grade). Diagnostic performance measures relate to the 35 women with an assignable Hecht grade; maternal and neonatal outcome comparisons (Tables 2–4) include all 40 women. PPV and NPV depend on the prevalence of high-grade disease in this cohort (71.4%) and are not transferable to populations with a different case mix.

Comparisons of continuous maternal and neonatal variables between low- and high-grade groups are presented in Table 2**.** Patients with high-grade placenta accreta spectrum experienced significantly greater estimated intraoperative blood loss compared with those with low-grade disease (median 2,500 mL [IQR 1650–3000] vs. 800 mL [IQR 600–2500]; *p* = 0.002).

**Table 2:** Comparison of continuous variables between Low and high grade groups.

Variable	Low grade (*n* = 15)	High grade (*n* = 25)	*p*-value
Blood loss (mL)	800 (600–2500)	2500 (1650–3000)	**0**.**002**
Gestational age (weeks)	35.2 (34.5–36.0)	34.0 (32.8–35.1)	**0**.**011**
Birth weight (g)	2550 (2300–2850)	2100 (1800–2600)	**0**.**014**
Apgar score at 5 min	9 (9–10)	8 (7–9)	**0**.**012**
BMI (kg/m^2^)^a^	26.4 (21.1–30.2)	22.1 (19.4–26.5)	0.061

Data are presented as median (interquartile range). *p* values from Mann–Whitney U test. BMI, body mass index.

a *n* = 14 in the low-grade group; one case excluded because of missing data.

Bold values indicate statistical significance.

Neonatal outcomes were less favorable in the high-grade group, including lower gestational age at delivery (median 34.0 weeks [IQR 32.8–35.1] vs. 35.2 weeks [IQR 34.5–36.0]; *p* = 0.011), lower birth weight (median 2,100 g [IQR 1800–2600] vs. 2,550 g [IQR 2300–2850]; *p* = 0.014), and lower Apgar score at 5 min (median 8 [IQR 7–9] vs. 9 [IQR 9–10]; *p* = 0.012).

Maternal body mass index did not differ significantly between grade groups (low grade median 26.4 kg/m^2^ [IQR 21.1–30.2] vs. high grade 22.1 kg/m^2^ [IQR 19.4–26.5]; *p* = 0.061).

Associations between histopathological grade and major surgical interventions are shown in Table 3. A statistically significant association was observed between high-grade PAS and the need for hysterectomy. All patients in the high-grade group (100%, *n* = 25) underwent hysterectomy, compared with 26.7% (*n* = 4) in the low-grade group (*p* < 0.001).

**Table 3:** Association of histological grade with surgical interventions.

Intervention	Category	Low Grade (*n* = 15)	High Grade (*n* = 25)	*p*-value
Hysterectomy	Performed	4 (26.7%)	25 (100%)	**< 0.001**
	Not Performed	11 (73.3%)	0 (0.0%)	
Arterial Ligation	Performed	3 (20.0%)	19 (76.0%)	**0.001**
	Not Performed	12 (80.0%)	6 (24.0%)	

Data are presented as *n* (%).

*P*-values were calculated using the Pearson Chi-Square test.

For the Hysterectomy analysis, Fisher's Exact Test is also appropriate due to the expected count in one cell being less than 5; its *p*-value is also < 0.001.

Bold values indicate statistical significance.

Similarly, internal iliac artery ligation was significantly more frequent in the high-grade group (76.0%, *n* = 19) than in the low-grade group (20.0%, *n* = 3; *p* = 0.001).

A trend toward increasing histopathological grade with a greater number of prior cesarean deliveries was observed (Table 4). The proportion of high-grade PAS increased from 37.5% in patients with no prior cesarean delivery to 77.8% in those with two or more previous cesareans. This trend did not reach statistical significance (*p* = 0.095).

**Table 4:** Association between number of previous cesarean sections and histological grade.

Previous CS	Low Grade (*n* = 15)	High Grade (*n* = 25)	Total (*N* = 40)
0 CS	5 (62.5%)	3 (37.5%)	8 (100%)
1 CS	8 (34.8%)	15 (65.2%)	23 (100%)
≥2 CS	2 (22.2%)	7 (77.8%)	9 (100%)

Data are presented as *n* (%). *P*-value from Chi-square test for trend = 0.095.

## 4 Discussion

This study suggests that prenatal ultrasound can help distinguish low- from high-grade PAS as defined by histopathological grading. Because 24 of the 25 high-grade cases were grade 3A, with a single grade 3D case and no grade 3E cases, our findings largely reflect grade 3A disease and should not be taken as representative of the full spectrum of high-grade Hecht pathology. Individual ultrasound signs showed different diagnostic characteristics. Myometrial thinning and placental lacunae had the highest sensitivity for high-grade disease, whereas placental bulging and bridging vessels showed higher, but only moderate specificity. Placental lacunae were frequent across the PAS spectrum and, despite high sensitivity, had limited value as an isolated predictor because of very low specificity. Several of these signs are not specific to invasive placentation. Myometrial thinning, loss of the clear zone, and placental bulging can all arise from a thin or dehiscent lower-segment scar under a placenta previa, without villous invasion. This overlap likely contributes to the modest specificity we observed. These findings support the use of a multiparametric ultrasound assessment rather than reliance on any single sign, but we did not test combinations of signs. The multiparametric approach we suggest is therefore an interpretation of the per-sign results, not a finding of this study, and would need a larger prospective cohort to confirm.

The observed diagnostic performance of ultrasound in this cohort is consistent with current literature. Previous studies and meta-analyses have shown that, when performed by experienced operators, prenatal ultrasound achieves high sensitivity and specificity for the detection of PAS (7, 9). Our results show that individual ultrasound markers demonstrate variable diagnostic performance in predicting histopathological grade, supporting the role of ultrasound in prenatal diagnosis and risk assessment of PAS. Our figures describe how well ultrasound separates histological grades, rather than detection of PAS. Higher histopathological grade was associated with substantially increased maternal morbidity. Patients with high-grade PAS experienced significantly greater intraoperative blood loss and required more complex surgical management, including hysterectomy and internal iliac artery ligation. At our institution, cesarean hysterectomy is the standard management strategy when extensive PAS is confirmed intraoperatively and placental separation is considered unsafe. Therefore, hysterectomy rates likely reflect not only histopathological grade itself but also institutional management strategy and intraoperative surgical judgment. Grades ≥3A also require a full-thickness specimen, which hysterectomy itself produces — so grade and hysterectomy are not independent, and their association is partly definitional. We read it as descriptive of this cohort rather than as evidence that grade determines the need for hysterectomy. These findings are in line with previous reports describing estimated blood loss in PAS ranging between 2,000 and 3,500 mL, particularly in cases with deeper placental invasion (10, 11).

Neonatal outcomes followed the same gradient. Gestational age at delivery was lower in high-grade cases (median 34.0 vs. 35.2 weeks, *p* = 0.011), as was birth weight (2,100 g vs. 2,550 g, *p* = 0.014). Earlier delivery in these cases was driven by maternal risk rather than fetal indication — a trade-off that makes accurate prenatal severity estimation consequential not only for surgical planning but for neonatal preparation as well.

The relationship between prior cesarean delivery and PAS risk and severity is well established (12, 13). In our cohort, a higher proportion of severe PAS was observed with an increasing number of prior cesarean deliveries; however, this association did not reach statistical significance.

### 4.1 Clinical implications

Accurate prenatal assessment of PAS grade has important clinical implications. Anticipation of high-grade disease enables appropriate multidisciplinary planning, selection of surgical approach, and preparation for major hemorrhage, as well as more informed patient counseling regarding the likelihood of hysterectomy (14). In our cohort, these risks were substantially higher in high-grade cases, supporting the use of a structured, multiparametric ultrasound approach as a tool for preoperative risk stratification. This remains a clinical inference rather than a tested strategy, since no composite score was evaluated here. Histopathological grade should not be equated with operative severity. Depth of invasion is only one determinant of surgical difficulty, alongside placental topography, pelvic vascularity, adjacent organ involvement, previous adhesions, and the experience of the operating team. Two cases of the same grade can differ considerably in operative risk.

### 4.2 Limitations

This study has several limitations. Its retrospective design and single-center setting may limit generalizability. These estimates apply to a selected, high-risk population referred to a tertiary center with specialist imaging and pathology resources, and should not be generalized to unselected obstetric populations or to settings without comparable expertise. The sample size was modest, which may have reduced statistical power, particularly for analyses of risk factors and delivery characteristics. Although ultrasound findings were documented before delivery and histopathology, sonographers had access to standard clinical information at the time of examination — including obstetric history, placental location, and prior magnetic resonance imaging when performed — and were therefore not blinded to clinical risk factors; this reflects routine practice. Sonographers’ knowledge of the clinical risk profile could favor recording signs as present. As nearly all referred women carried risk factors, this is unlikely to have differed greatly between grade groups, so any overestimation is probably limited; residual bias cannot, however, be fully excluded. The reference standard was also not uniform — Hecht grading was published in 2020 but recruitment began in 2018, so earlier cases were graded retrospectively and later ones at routine sign-out, and some misclassification cannot be excluded. Additionally, PAS is a histologically heterogeneous disease, and even targeted sampling of the area of deepest placental invasion cannot guarantee that the most invasive focus is captured in every case; isolated foci of deeper invasion could therefore have been underrepresented. Grading in women managed without hysterectomy relied on partial myometrial resection specimens, in which the full thickness of the uterine wall is not available; the depth of invasion assessable in these specimens is therefore more limited than in hysterectomy specimens, which may have affected the depth-based comparison between groups. A further inconsistency in the reference standard is that five cases were classified as BPMF rather than assigned a PAS grade, reflecting the material available in conservatively managed pregnancies. The Hecht system is also a pathological classification and does not capture topographic features — bladder, parametrial, or cervical involvement, and vascular anatomy — that materially influence surgical complexity; cases of similar histological grade may therefore differ substantially in operative risk. Finally, while histopathological grading provides an objective reference standard, it is only available postnatally, limiting its direct applicability to prenatal decision-making.

## 5 Conclusion

Standardized prenatal ultrasound assessment discriminated low- from high-grade placenta accreta spectrum as defined by Hecht histopathological grading. Higher histological grade was associated with greater blood loss, more frequent hysterectomy, and less favorable neonatal outcomes in this cohort — differences that argue against treating PAS as a single surgical problem. Incorporating standardized ultrasound evaluation into multidisciplinary PAS pathways provides a practical basis for surgical planning and for counseling patients about the likelihood of hysterectomy and hemorrhagic morbidity. These are single-center results, and the assignable grade depended partly on the available specimen. Grade reflects one determinant of operative risk rather than overall severity. These findings apply mainly to grade 3A disease, as grades 3D and 3E were rare in this cohort, and the multiparametric approach needs confirmation in larger multicenter studies.

## Data Availability

The datasets presented in this study can be found in online repositories. The names of the repository/repositories and accession number(s) can be found below: https://doi.org/10.6084/m9.figshare.31460218.
