# Supplementary material for: Diagnostic performance of standardized prenatal ultrasound signs for predicting Hecht histopathological grade in placenta accreta spectrum: a retrospective study
Source: Front Med (Lausanne). 2026 Sep 18;13:1933234. doi: 10.3389/fmed.2026.1933234 (PMC13630943; doi:10.3389/fmed.2026.1933234)
Supplement: Supplementary file 1 [file Table1.docx]

**Supplementary Table S1.** Diagnostic performance of prenatal ultrasound signs for identifying high-grade placenta accreta spectrum, restricted to the 35 women with an assignable Hecht grade (10 low-grade, 25 high-grade). Values are percentages with 95% Wilson score confidence intervals. Figures from the primary analysis (n = 40, including five cases of basal plate myometrial fibers in the low-grade group) are shown in parentheses for comparison.

| Ultrasound sign | Sensitivity, % (95% CI) | Specificity, % (95% CI) | PPV, % (95% CI) | NPV, % (95% CI) |
| --- | --- | --- | --- | --- |
| Myometrial thinning (<1 mm) | 92.0 (75.0–97.8) *(92.0)* | 30.0 (10.8–60.3) *(46.7)* | 76.7 (59.1–88.2) *(74.2)* | 60.0 (23.1–88.2) *(77.8)* |
| Loss of the clear zone | 84.0 (65.3–93.6) *(84.0)* | 50.0 (23.7–76.3) *(60.0)* | 80.8 (62.1–91.5) *(77.8)* | 55.6 (26.7–81.1) *(69.2)* |
| Placental lacunae | 92.0 (75.0–97.8) *(92.0)* | 20.0 (5.7–51.0) *(13.3)* | 74.2 (56.8–86.3) *(63.9)* | 50.0 (15.0–85.0) *(50.0)* |
| Bridging vessels | 80.0 (60.9–91.1) *(80.0)* | 70.0 (39.7–89.2) *(73.3)* | 87.0 (67.9–95.5) *(83.3)* | 58.3 (32.0–80.7) *(68.8)* |
| Abnormal placental–uterine interface | 80.0 (60.9–91.1) *(80.0)* | 50.0 (23.7–76.3) *(53.3)* | 80.0 (60.9–91.1) *(74.1)* | 50.0 (23.7–76.3) *(61.5)* |
| Placental bulging | 80.0 (60.9–91.1) *(80.0)* | 70.0 (39.7–89.2) *(73.3)* | 87.0 (67.9–95.5) *(83.3)* | 58.3 (32.0–80.7) *(68.8)* |

CI, confidence interval; NPV, negative predictive value; PPV, positive predictive value.

Sensitivity is unchanged from the primary analysis because the high-grade group (n = 25) is unaffected by the exclusion. Prevalence of high-grade disease rises from 62.5% to 71.4%, which accounts for the higher positive predictive values and lower negative predictive values.
